# PO-llution control: a cross-sectional study on the role of antimicrobial stewardship in reducing healthcare’s carbon footprint

**DOI:** 10.1093/jacamr/dlaf146

**Published:** 2025-08-28

**Authors:** Saied Ali, Sadhbh Gash, Niamh Weir, Karen Burns, Binu Dinesh, Helene Mcdermott, Fidelma Fitzpatrick, Sinead O’Donnell, Ciara O’Connor

**Affiliations:** Department of Clinical Microbiology, Beaumont Hospital, Dublin 9, Ireland; Department of Clinical Microbiology, Royal College of Surgeons in Ireland, Dublin 2, Ireland; Department of Pharmacy, Beaumont Hospital, Dublin 9, Ireland; Department of Pharmacy, Beaumont Hospital, Dublin 9, Ireland; Department of Clinical Microbiology, Beaumont Hospital, Dublin 9, Ireland; Department of Clinical Microbiology, Royal College of Surgeons in Ireland, Dublin 2, Ireland; Department of Clinical Microbiology, Beaumont Hospital, Dublin 9, Ireland; Department of Clinical Microbiology, Beaumont Hospital, Dublin 9, Ireland; Department of Clinical Microbiology, Beaumont Hospital, Dublin 9, Ireland; Department of Clinical Microbiology, Royal College of Surgeons in Ireland, Dublin 2, Ireland; Department of Clinical Microbiology, Beaumont Hospital, Dublin 9, Ireland; Department of Clinical Microbiology, Royal College of Surgeons in Ireland, Dublin 2, Ireland; Department of Clinical Microbiology, Beaumont Hospital, Dublin 9, Ireland

## Abstract

**Objectives:**

To evaluate the environmental impact of prolonged IV antimicrobial courses and identify opportunities for improved antimicrobial stewardship (AMS) practices.

**Methods:**

A retrospective cross-sectional study was conducted using AMS ward-round data from January 2023 to December 2024 at a tertiary hospital in Dublin, Ireland. Data on IV antimicrobial prescriptions, AMS recommendations for discontinuation or IV to oral switch (IVOS) and prescriber acceptance were reviewed. A life cycle assessment, informed by published literature, was used to estimate the carbon footprint associated with IV use.

**Results:**

Of 1929 antimicrobial prescriptions reviewed, 58% (*n* = 1119) were being administered IV. Among 435 IV prescriptions with AMS, recommendations to stop (*n* = 357) or IVOS (*n* = 78), 229 (52.6%) were accepted, resulting in a reduction of 106.5 kg of clinical waste and 261.2 kg carbon dioxide equivalents (CO₂e) emissions. The remaining 206 IV prescriptions (47.4%) were categorized as prolonged IV prescriptions, generating 98.8 kg of clinical waste and 245.8 kg CO₂e; averaging 0.48 kg of waste and 1.19 kg CO₂e per prescription. To contextualize, the carbon footprint of each prolonged prescription equates to driving 6.2 km, performing 10 chest X-rays or operating a 10 W light-emitting diode bulb continuously for 1200 h. Piperacillin–tazobactam, amoxicillin–clavulanic acid, cefuroxime, metronidazole and meropenem together accounted for over 84% of total emissions, with piperacillin–tazobactam alone contributing 97.5 kg CO₂e and 41.6 kg of waste from 62 prolonged prescriptions.

**Conclusions:**

In addition to patient safety risks, prolonged IV antimicrobial courses generate considerable environmental waste. Aligning AMS with sustainability goals may contribute to addressing the dual crises of antimicrobial resistance and climate change.

## Introduction

Antimicrobial stewardship (AMS) plays a critical role in optimizing antimicrobial use, improving patient outcomes and reducing the emergence and spread of antimicrobial resistance (AMR).^1,2^ One of the most impactful, but under-utilized, AMS interventions is the timely transition from IV to oral (PO) antimicrobials (IVOS).^3–6^ This practice, when clinically appropriate, not only contributes to reduced risk of complications, such as intravascular catheter-associated infections, but also supports resource efficiency and shorter hospital stays.^2^ Furthermore, many commonly used antimicrobials, including metronidazole, ciprofloxacin and linezolid, exhibit excellent oral bioavailability (often  ≥ 90%), enabling reliable PO therapy once the patient is clinically stable.^7^ Despite these well-established benefits, adherence to IVOS guidelines remains inconsistent, with many patients continuing on IV antimicrobials beyond the necessary duration.

Beaumont Hospital has a long-running AMS programme for over two decades, delivered through the clinical microbiology inpatient consult service, which has expanded significantly over recent years. A continued emphasis has been placed on improving compliance with IVOS. Under the leadership of consultant clinical microbiologists and antimicrobial pharmacists, the multi-disciplinary AMS team has introduced structured prescribing indicators, increased the frequency of ward-based AMS rounds and strengthened prescriber education.^8^ These efforts have led to more frequent AMS-led interventions and a gradual improvement in prescribing practices. However, local audit data from 2023 and 2024 demonstrate missed opportunities for IVOS, revealing an important area for quality improvement.

Beyond patient safety and antimicrobial optimization, delayed IVOS has far-reaching implications for environmental sustainability, one element of Beaumont Hospital’s newly developed Strategic Plan 2025–2030.^9^ Under Strategic Priority 07: *Our Performance, Productivity and Sustainability*, the hospital explicitly commits to ‘minimize our environmental impact through sustainable practices’ and to ‘optimize resource utilization to enhance productivity and value’. As part of this commitment, Beaumont Hospital is actively progressing towards *My Green Lab* certification, reflecting an institutional effort to integrate internationally recognized sustainability standards into routine clinical and laboratory practice.^10^

Unnecessary use of the IV route consumes a disproportionate amount of materials and energy. Single-use plastics such as IV tubing, saline bags, syringes and protective packaging accumulate rapidly, contributing significantly to healthcare waste streams. In addition, the production, distribution and disposal of IV antimicrobials require energy and material resources, with each stage contributing to a higher overall carbon footprint compared with oral formulations.^11^

Each additional day of avoidable IV administration represents not only a missed clinical stewardship opportunity but also an avoidable environmental burden. In a high-volume tertiary centre such as Beaumont—which delivers over 71 000 day-procedures and 28 000 inpatient admissions annually—the cumulative environmental cost of suboptimal prescribing is substantial. As hospitals increasingly seek to align clinical practice with planetary health principles, antimicrobial prescribing must evolve to reflect both therapeutic and environmental responsibilities.

This study focuses on quantifying the environmental impact of delayed IVOS, framing it as both an AMS and a sustainability issue. By identifying prescribing patterns, estimating avoidable plastic and pharmaceutical waste and aligning findings with the hospital’s strategic objectives, this research aims to generate actionable evidence that supports further implementation of environmentally responsible AMS practices.

## Materials and methods

### Ethics

Ethical approval was not required as this is a retrospective study of an anonymized dataset with neither human participants nor animals.

### Study setting

Beaumont Hospital is an adult tertiary referral centre located in Dublin, Ireland, providing tertiary and quaternary care across 54 medical specialties. It serves as a national centre for renal transplantation, neurosurgery and cochlear implantation and a regional centre for cancer care, operating over 820 inpatient beds.^12^ There is no electronic prescribing system in Beaumont Hospital; all prescribing is done using paper-based medicines prescription and administration records (MPARs).

### AMS team overview

The AMS service is provided by a multi-disciplinary team comprising consultant and specialist trainee clinical microbiologists and antimicrobial pharmacists. The team conducts daily rounds in all critical care units, thrice-weekly AMS rounds across medical and surgical wards, along with a weekly targeted AMS round for patients with *Clostridioides difficile* infection (CDI), promoting appropriate antimicrobial use through detailed patient reviews, real-time prescriber feedback and guideline-driven interventions. To inform AMS recommendations, the multi-disciplinary team conducts a systematic review of each patient’s clinical notes, microbiology and biochemistry results, radiology findings and relevant guidelines ensuring that their recommendations are individualized, patient specific and evidence based.

In parallel, the AMS team delivers regular education to clinicians, pharmacists, nurses and students to embed AMS principles into daily practice. To further raise awareness, AMS-led campaigns utilize digital screensavers, intranet messages and seasonal initiatives highlighting key AMS issues and guideline updates. The team also manages Beaumont’s Antimicrobial Guidelines App, a crucial point-of-care tool updated regularly to support clinical decision-making. Through defined key performance indicators (KPIs), the AMS team monitors prescribing trends and contributes to hospital audits, ensuring compliance with national standards.^13,14^

### Study design

A retrospective analysis of antimicrobial prescribing practices over 2 years, 2023–24 inclusive, was conducted using data collected from the routine AMS ward rounds undertaken in medical and surgical wards.

The focus of this analysis was to identify instances in which IV antimicrobials were not stopped or switched to PO despite an AMS team recommendation. Each case of non-compliance with an IVOS or stop recommendation was categorized as a prolonged IV antimicrobial prescription. The specific antimicrobial was recorded to facilitate a detailed evaluation of prescribing patterns and associated environmental impact.

### Calculation of carbon dioxide equivalents

To quantify the environmental impact associated with prolonged IV antimicrobial prescriptions for specific formulations used at our institution, a life cycle assessment (LCA) was undertaken, guided by the PAS 2050:2011 standard.^15–18^ This framework enables the comprehensive evaluation of greenhouse gas emissions associated with a product or service throughout its entire life cycle—from raw material extraction to end-of-life disposal. In this study, the LCA was applied to assess the full carbon footprint of IV antimicrobial administration in a hospital setting, aiming to highlight the often-overlooked environmental consequences of unnecessary antimicrobial use.

The assessment began with the estimation of embodied carbon linked to the production and packaging of antimicrobial drugs and the accompanying single-use medical consumables required for administration. These included syringes, IV giving sets, drug vials, alcohol wipes, gloves and saline flushes. Each item was weighed, and its material composition was recorded to determine the quantity and type of waste generated during one routine day of IV drug administration. Emission factors were obtained from the Inventory of Carbon and Energy (ICE) database, which provides standardized values for the carbon emissions associated with the manufacture of various materials, including plastics, glass and paperboard.^19^ This allowed for a detailed calculation of the emissions embedded in the materials themselves.

Transport emissions were then estimated based on the distance between the site of drug manufacture or distribution and the healthcare facility. Assumptions regarding transport mode (primarily road freight) and distance were used in combination with published emission coefficients to estimate the additional carbon burden introduced by delivery logistics.^20^

Following administration, the waste generated was characterized, and its disposal pathway was assessed. Emissions associated with waste disposal were calculated using data from the Greener National Health Service programme, which provides specific carbon factors for various waste streams, including clinical incineration and landfill.^21^ By categorizing the waste and applying the appropriate disposal emission factors, downstream environmental impacts were effectively incorporated into the total LCA.

All emission data were ultimately expressed in terms of carbon dioxide equivalents (CO₂e), enabling standardized comparison across each life cycle stage. The final outcome was a cumulative estimate of the total CO₂e generated per 1 day of routine IV antimicrobial prescription.

See Appendix (available as Supplementary data at *JAC-AMR* Online) for a detailed explanation of the LCA calculation, including a worked example.

### Statistical analysis

Descriptive statistics were used to summarize antimicrobial prescribing patterns, IVOS outcomes, instances of prolonged IV antimicrobial prescription and associated CO₂e emissions. Continuous variables, such as estimated CO₂e emissions, were expressed as medians with IQRs. Categorical variables, including prescribing outcomes (e.g. IV continued versus stopped and advice accepted versus declined), were summarized as frequencies and percentages. To compare proportions between groups—such as between the medical and surgical patients—chi-square tests were used for larger sample sizes, while Fisher’s exact test was applied where counts were small. A significance level of *P* < 0.05 was used to determine statistical significance.

## Results

As shown in Table 1, 1929 antimicrobial prescriptions (*n* = 1116; 57.9% IV) were reviewed: 63.1% (*n* = 1217) in medical and 36.9% (*n* = 712) in surgical patients. The proportion of IV prescriptions was higher in medical than surgical patients (*n* = 615; 55.1% versus *n* = 501; 44.9%).

**Table 1.** Antimicrobial prescribing and stewardship outcomes by patient group

	Total, *n* (%)	Medical, *n* (%)	Surgical, *n* (%)
Antimicrobial prescriptions reviewed	1929	1217 (63.1%)	712 (36.9%)
IV antimicrobials prescribed	1116 (57.9%)	615 (55.1%)	501 (44.9%)
Recommendation to stop IV antimicrobials	357 (32%)	141 (22.9%)	216 (43.1%)
Advice accepted	189 (52.9%)	67 (47.5%)	122 (56.5%)
Advice declined	168 (47.1%)	74 (52.5%)	94 (43.5%)
Recommendation for IVOS	78 (7%)	29 (4.7%)	49 (9.8%)
Advice accepted	40 (51.3%)	13 (44.8%)	27 (55.1%)
Advice declined	38 (48.7%)	16 (55.2%)	22 (44.9%)

Almost one-third of IV prescriptions (*n* = 357; 32%) were deemed unnecessary, with a recommendation to stop given. A recommendation to stop was more common for surgical than medical patients (*n* = 216; 43.1% versus *n* = 141; 22.9%). The recommendation to stop was accepted in 189 cases (52.9%) with a higher acceptance in surgical (56.5%; *n* = 122) than in medical patients (47.5%; *n* = 67). However, this difference was not statistically significant (chi-square, *P* = 0.12).

Of the IV prescriptions in surgical patients, 52 (10.4%) were for surgical antimicrobial prophylaxis (SAP) and 449 (89.6%) for therapy. A recommendation to stop was given for 82.7% of SAP and 38.5% of therapy prescriptions. Acceptance of this recommendation was significantly lower for SAP (18.6%) compared with therapy (65.9%) (chi-square = 29.44; *P* < 0.0001).

There were 78 (7%) IV prescriptions with an IVOS recommendation given (49 in surgical and 29 in medical patients). The recommendation for IVOS was accepted in 40 cases (51.3%), again with a higher acceptance in surgical (55.1%; *n* = 27) than in medical patients (44.8%; *n* = 13). Fisher’s exact test revealed no statistically significant difference in acceptance rates (*P* = 0.48).

Overall, among 435 IV prescriptions reviewed with AMS recommendations to discontinue therapy or IVOS, 229 (52.6%) were accepted. This corresponded to an estimated reduction of 106.5 kg of clinical waste and 261.2 kg CO₂e emissions, as shown in Table 2. Conversely, there were 206 IV prescriptions (47.4%) where AMS advice was declined, and these were categorized as prolonged IV antimicrobial prescriptions. The total estimated clinical waste generated from these non-compliant cases was 98.8 kg, with an associated cumulative carbon footprint of 245.8 kg CO₂e. On average, each prolonged IV prescription resulted in 0.48 kg of waste (approximately the weight of a standard loaf of bread) and 1.19 kg CO₂e in emissions (equating to driving a standard automatic vehicle for 6.2 km, performing 10 chest X-rays or operating a 10 W light-emitting diode (LED) light bulb continuously for 1200 h).

**Table 2.** Impact of AMS recommendation acceptance on estimated waste and carbon emissions from prolonged IV antimicrobial prescriptions, *n* = 435

	Total, *n* (%)	Waste generated, kg	Carbon emissions generated, kg CO₂e
AMS recommendation accepted	229 (52.6)	106.5	261.2
AMS recommendation declined	206 (47.4)	98.8	245.8

**Figure 1.**
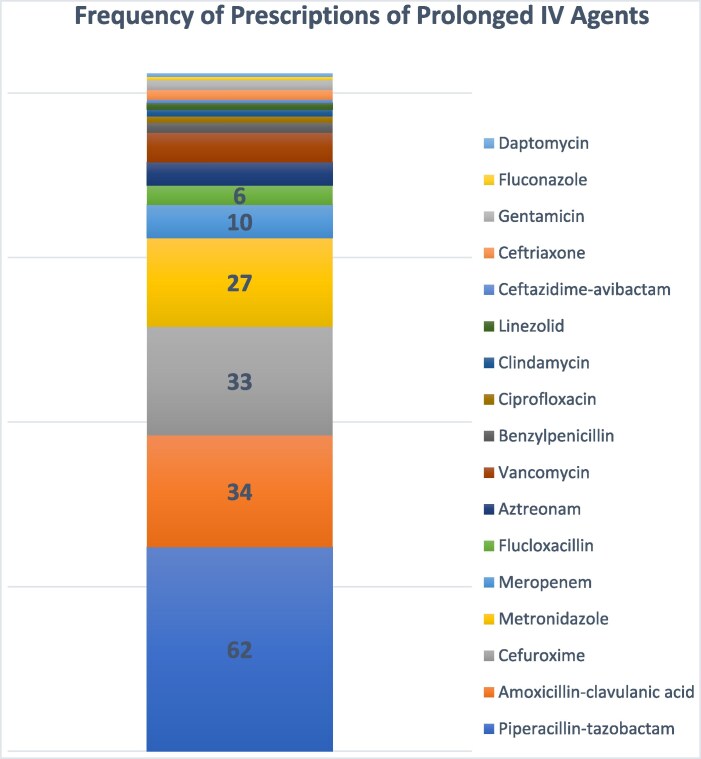
Stacked bar graph showing distribution of antimicrobials associated with frequency of prolonged IV antimicrobial prescriptions.

**Figure 2.**
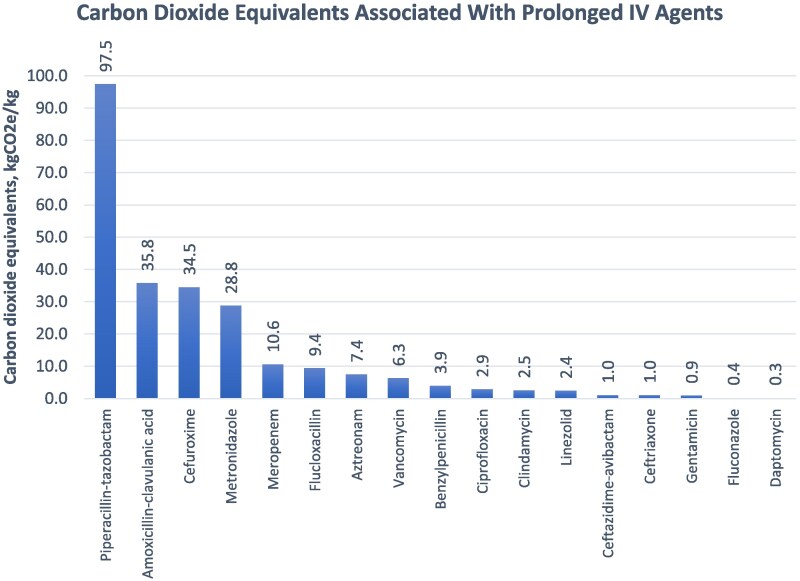
CO₂e associated with prolonged IV antimicrobial prescriptions over the study period.

Among the 17 distinct antimicrobial agents evaluated (Figures 1 and 2), piperacillin–tazobactam accounted for the largest environmental burden, contributing 97.5 kg CO₂e and 41.6 kg of waste across 62 prolonged prescriptions. This was followed by amoxicillin–clavulanic acid (35.8 kg CO₂e), cefuroxime (34.5 kg CO₂e), metronidazole (28.8 kg CO₂e) and meropenem (10.6 kg CO₂e). Collectively, these five agents were responsible for over 84% of the total carbon emissions observed during the study period.

Several agents with excellent PO bioavailability, including ciprofloxacin, metronidazole and linezolid, were administered for prolonged courses. Together, these three agents accounted for 34.0 kg CO₂e, representing nearly 14% of the total emissions observed. While metronidazole alone contributed 28.8 kg CO₂e across 27 prescriptions, ciprofloxacin and linezolid added a combined 5.2 kg CO₂e from just four cases.

Based upon the 206 non-compliant cases from a total of 1929 antimicrobial prescriptions reviewed, when standardized to every 100 prescriptions reviewed, approximately 10.7 cases resulted in prolonged IV antimicrobial use, despite an AMS recommendation to stop or IVOS. The environmental impact of the 206 cases reviewed amounted to 245.8 kg of CO₂e and 98.8 kg of clinical waste. From this, the average emission per non-compliant case was calculated at 1.19 kg CO₂e, and average waste at 0.48 kg, which was then applied to the 10.7 case rate to estimate emissions and waste per 100 prescriptions. This resulted in an estimated 12.7 kg of CO₂e and 5.1 kg of waste generated for every 100 prescriptions reviewed.

## Discussion

This study demonstrates the environmental burden of unnecessary IV antimicrobial use. The role of healthcare in contributing to climate change is increasingly under scrutiny, with hospitals accounting for approximately 4.4% of global greenhouse gas emissions.^22,23^ In this context, AMS not only remains a vital strategy aimed at ensuring the responsible use of antimicrobials to improve patient safety, reduce AMR and promote efficient use of healthcare resources but also provides a tangible way to decarbonize elements of care delivery. Integrating environmental metrics into AMS audits and discussions enhances their relevance within the global health and sustainability agenda.^1^

A central element of AMS is the timely and clinically appropriate transition from IV to PO antimicrobials. When implemented effectively, this switch allows patients to switch from the IV route sooner, which in turn supports earlier discharge, improves overall comfort and lowers the risk of complications such as intravascular catheter-related infections, restricted mobility and delayed rehabilitation.^24,25^

Additionally, prolonged IV administration intensifies the workload for nursing staff, who are responsible for IV preparation, line maintenance, infusion monitoring and documentation. This unnecessary demand diverts nursing time away from other critical aspects of patient care, placing avoidable strain on already stretched healthcare teams.^26^ Clinical pharmacists and AMS teams also experience an added burden, dispensing, reviewing and managing prescriptions that could be simplified through use of PO alternatives.^27,28^

In this study of 1929 antimicrobial prescriptions reviewed over 2 years, 57.9% were IV, and 39% were flagged as unnecessary, with either a stop or IVOS recommendation given. However, only half of these recommendations were accepted. We recognize that patient-specific factors not fully documented in our dataset may have influenced decisions to continue IV therapy. However, given the rigorous multi-disciplinary assessment process, we consider the impact of such undocumented factors on our overall findings to be minimal.

Notwithstanding the obvious potential financial and labour cost savings associated with stopping unnecessary antimicrobial prescriptions and early IVOS, prescriber habits, clinical inertia and risk aversion often lead to prolonged or unnecessary IV prescriptions. These behaviours may be reinforced by hierarchical dynamics, reluctance to challenge senior decisions or perceived patient expectations.^29–31^ Further compounding these challenges, IVOS is not always a clear-cut substitution; it often necessitates nuanced clinical decision-making and consultation with clinical microbiology or infectious disease specialists or adherence to guidelines—factors that may contribute to prescriber hesitation.^7^

Additionally, AMS engagement may vary across specialties. Clinicians in medical specialties may adopt a more individualized prescribing approach to prescribing, whereas surgeons appear more responsive to structured AMS recommendations; however, these observed trends did not reach statistical significance (*P* > 0.05).^30,31^

However, while discontinuation was advised for 82.7% of SAP prescriptions, only 18.6% were stopped, compared with 65.9% of therapy prescriptions (*P* < 0.0001). Prolonged SAP beyond the recommended post-operative window is a well-established risk factor for AMR, *C. difficile* infection and increased healthcare costs, without evidence of benefit in preventing surgical site infections.^32,33^ This high prevalence of prolonged SAP reflects a wider national trend. The 2024 Irish point prevalence survey reported that 26.0% of SAP prescriptions extended beyond 24 h, representing a gradual improvement from 29.5% in 2023, yet remaining above the national target of 22%.^34^ At Beaumont Hospital, the rate of SAP prolongation has historically exceeded national averages, reaching 58.4% in 2023.^34^ Contributing factors include the frequent documentation of stop dates only within operative notes rather than transcription to the MPAR, which limits visibility for prescribers, nursing staff and pharmacists. Additionally, habitual prescribing practices, perceived medico-legal concerns and persistent misconceptions regarding the benefits of extended SAP contribute to unnecessary prolongation.^35,36^ These findings highlight the need for robust, system-level interventions and have prompted the launch of a new Antimicrobial Resistance and Infection Control (AMRIC) initiative aimed at reducing excessive SAP duration across Irish hospitals.^37^

Overcoming these barriers requires not only education and system-level supports but also cultural change. AMS does not rest solely on prescribers. A more collaborative, multi-disciplinary approach that empowers nursing staff and even engages patients themselves is essential for success.^38^ Nurses are often the first to observe patient readiness for PO medications and, with appropriate training and support, can act as key advocates for initiating the conversation around timing of IVOS.^39^ Similarly, patients who are educated about the safety and potential benefits of the PO route may be more likely to question unnecessary IV use and participate in their care decisions.^40^ Embedding stewardship awareness across all levels of care helps build a proactive, safety-focused culture and increases the likelihood that AMS interventions will be sustained.^41^

Across 206 cases of prolonged IV therapy, an estimated 98.8 kg of clinical waste and 245.8 kg CO₂e were generated. Each non-compliant case resulted in an average of 1.19 kg CO₂e and 0.48 kg of waste. A small number of antimicrobials, particularly piperacillin–tazobactam, accounted for the majority of these emissions, followed by agents such as amoxicillin–clavulanic acid, cefuroxime and metronidazole. Strikingly, some of these antimicrobials, such as metronidazole, ciprofloxacin and linezolid, have excellent oral bioavailability, highlighting easy wins for environmentally responsible care.

When standardized to every 100 prescriptions reviewed, approximately 10.7 cases of prolonged IV use were observed, translating to an estimated 12.7 kg of CO₂e and 5.1 kg of waste. These measurable outputs provide a compelling benchmark for assessing the environmental cost of stewardship non-compliance and offer a unique opportunity to connect clinical decisions with their broader ecological impact.

AMS ward rounds at our institution are conducted three times weekly across medical and surgical wards, and data from these rounds constituted the basis of this study. As not all inpatients receiving antimicrobials are reviewed during these rounds, some patients may have been missed, potentially resulting in an underestimation of the total environmental impact of prolonged IV therapy. In addition, the absence of electronic prescribing at our institution limits automated and timely AMS interventions. Implementation of electronic prescribing, which can often incorporate decision support tools for prescribers, has been shown to significantly improve adherence to IVOS protocols and reduce inappropriate prescribing.^42^ In Ireland, a national AMS IVOS tool kit has been produced, but broader implementation and national data transparency remain limited.^14^ Strengthening these systems could support more consistent stewardship across hospitals and healthcare networks.

Although this study focuses on inpatient secondary care, the findings have broader relevance across healthcare settings. In virtual wards and outpatient parenteral antimicrobial therapy (OPAT) programmes, where complex care is increasingly delivered in community settings, the minimization of unnecessary IV therapy remains crucial. Reducing inappropriate IV therapy in these settings reduces both clinical risks and the environmental burden associated with home-based infusion equipment, packaging and staff travel.^43^ Similarly, in primary care, where IV antimicrobials are not routinely used, the selection of oral agents with narrower spectra and shorter durations is a key stewardship principle for reducing prescribing-related emissions. Oral antimicrobials generally carry a lower carbon and waste footprint than IV formulations; however, inappropriate or extended courses still contribute to environmental harm through emissions from pharmaceutical production, distribution and waste disposal.^44–46^ Additionally, patient travel for primary care consultations contributes further to the overall environmental footprint associated with antimicrobial use.^47,48^

Finally, linking AMS to broader sustainability goals may offer an additional motivator for behaviour change. Framing AMS not only as a patient safety or cost reduction measure but also as a climate action initiative may resonate with a wider audience and enhance both clinician and patient engagement. Campaigns such as the World Health Organization’s World AMR Awareness Week serve as critical platforms to reinforce these connections.^49^

AMS is not just about combating AMR—it is about delivering safer, more efficient and more sustainable healthcare. Unnecessarily prolonged IV courses and delays in IVOS represent missed opportunities for reducing both clinical harm and carbon emissions. As hospitals confront the dual challenges of AMR and climate change, AMS programmes must evolve to reflect both therapeutic and planetary health priorities. Strengthening collaboration among prescribers, nurses and patients—while leveraging digital tools and sustainability messaging—can help transform AMS into a model of efficient and future-facing care.

## Supplementary Material

dlaf146_Supplementary_Data

## Supplementary data

Appendix is available as Supplementary data at *JAC-AMR* Online.
