# Supplementary material for: PO-llution control: a cross-sectional study on the role of antimicrobial stewardship in reducing healthcare’s carbon footprint
Source: JAC Antimicrob Resist. 2025 Aug 28;7(4):dlaf146. doi: 10.1093/jacamr/dlaf146 (PMC12391753; doi:10.1093/jacamr/dlaf146)
Supplement: dlaf146_Supplementary_Data [file dlaf146_supplementary_data.docx]

**APPENDIX**

**Detailed Methodology for Life Cycle Assessment (LCA)**

A LCA was performed to estimate the carbon footprint associated with prolonged intravenous (IV) antimicrobial use. The LCA was conducted in accordance with PAS 2050:2011 guidelines and the Greenhouse Gas (GHG) Protocol Product Life Cycle Accounting and Reporting Standard, providing a comprehensive assessment of greenhouse gas emissions across all major life cycle stages. Emissions are expressed as carbon dioxide equivalents (CO₂e).

The LCA included three principal stages:

1. Purchased goods and services

This stage accounted for the upstream emissions from manufacturing and packaging of IV antimicrobials and all associated single-use consumables.

Emission factors for formulation packaging were obtained from the Embodied Carbon Footprint Database (Circular Ecology) and the Inventory of Carbon and Energy (ICE) databases. For each antimicrobial, the material composition of vials, stoppers and external packaging was considered. We included IV giving sets, syringes, saline flushes, alcohol swabs and gloves. Individual item weights were measured and material types were recorded (e.g., paper, glass, rubber).

For example, considering a single dose of IV amoxicillin-clavulanic acid; each vial contained approximately 34.28 g of glass and the IV giving set comprised 71.63 g of plastic, 11.77 g of paper and 15.22 g of rubber. All weights were converted to kilograms for calculation purposes.

Emission factors used were 1.2 kgCO₂e per kg for glass, 3.1 kgCO₂e per kg for plastic, 0.9 kgCO₂e per kg for paper and 2.9 kgCO₂e per kg for rubber. The sum of the products of each material weight and its corresponding emission factor yielded the total embodied carbon for manufacturing and packaging a single dose; which in the case of amoxicillin-clavulanic acid amounted to 0.32 kgCO₂e per dose.

For each prescription, the cumulative embodied carbon from all items used over a typical daily IV administration cycle was calculated and then aggregated over the study period. As amoxicillin-clavulanic acid is typically administered 8-hourly (three doses per day), this resulted in an estimated 0.95 kgCO₂e per day. Over the study period, there were 34 instances of non-compliance with AMS recommendations for prolonged IV use of amoxicillin-clavulanic acid, resulting in a total estimated carbon emission of 32.4 kgCO₂e.

All subsequent worked examples in this Appendix are based on these 34 instances of non-compliance with AMS recommendations for prolonged IV use of amoxicillin-clavulanic acid.

1. Transportation and distribution

This stage captured emissions arising from the distribution of antimicrobials and associated consumables from manufacturing sites to the hospital. Emission estimates were calculated using the CarbonCare online emissions calculator (<https://www.carboncare.org/en/co2-emissions-calculator>). The mass of pharmaceuticals and their packaging was weighed and incorporated into the calculations to ensure accuracy. Transport routes were determined based on the supplier, either B. Braun Medical Limited in the United Kingdom or United Drug in Ireland. For international shipments, emissions were modelled to include sea transport followed by road delivery to Beaumont Hospital.

In the case of IV amoxicillin-clavulanic acid, this distribution stage contributed approximately 0.3 kgCO₂e per kilogram per dose, translating to 0.9 kgCO₂e per day for an 8-hourly dosing regimen and a cumulative total of 3.06 kgCO₂e across the study period.

1. Waste management

All waste associated with IV antimicrobial administration was classified as healthcare-associated clinical waste requiring high-temperature incineration. Emission factors for incineration were sourced from the Greener NHS Carbon Footprint Plus program and Irish waste data, with a standard emission factor of approximately 0.021 kgCO₂e per kilogram applied regardless of the material. Similar to the manufacturing stage, the weight of waste generated per prescription was measured and multiplied by the abovementioned emission factor associated with incineration to estimate disposal-related emissions.

Considering a single dose of IV amoxicillin-clavulanic acid, which generated approximately 0.13 kg of waste, multiplication by the emission factor resulted in 0.0028 kgCO₂e per dose. This equated to 0.0084 kgCO₂e per day for an 8-hourly dosing schedule and a cumulative total of 0.28 kgCO₂e over the study period.

Total emissions

For each IV antimicrobial prescription the total emissions (kgCO₂e) were calculated as:

Total CO₂e = Manufacturing and Packaging Emissions + Transport Emissions +

Waste Disposal Emissions

For amoxicillin-clavulanic acid, the total emissions, i.e., combining manufacturing, transport and waste disposal stages, were therefore estimated at 0.35 kgCO₂e per dose, 1.05 kgCO₂e per day (based on an 8-hourly dosing regimen), and a cumulative total of 35.7 kgCO₂e over the study period.

The same process was applied to other antimicrobials.
